# Supplementary material for: The genome sequence of the sand star, Astropecten irregularis (Pennant, 1777)
Source: Wellcome Open Res. 2024 Aug 7;9:431. [Version 1] doi: 10.12688/wellcomeopenres.22821.1 (PMC11480709; doi:10.12688/wellcomeopenres.22821.1)
Supplement: Peer review history

# Reviewer report: Reviewer response for version 1

**DOI:** 10.21956/wellcomeopenres.25127.r95804

**Published:** 2024-10-15

**Authors:** Ferdinand Marlétaz

**Affiliations:** 1 Centre for Life’s Origin & Evolution, Department of Ecology, Evolution & Environment, University College London, London, UK

This report described the sequencing and assembly of the sea star *Astropecten irregularis* at chromosome scale. It appears accurate and well-described. I will reiterate some usual comments I make on such genome notes: the HiC contact map appears difficult to read, and the HiC protocol is not sufficiently detailed. Also,  the sex of the specimen used for assembly is not specified, it is a pity.

Are sufficient details of methods and materials provided to allow replication by others?

Partly

Is the rationale for creating the dataset(s) clearly described?

Yes

Are the datasets clearly presented in a useable and accessible format?

Yes

Are the protocols appropriate and is the work technically sound?

Yes

Reviewer Expertise:

Genomics, phylogeny, Zoology

I confirm that I have read this submission and believe that I have an appropriate level of expertise to confirm that it is of an acceptable scientific standard.

# Reviewer report: Reviewer response for version 1

**DOI:** 10.21956/wellcomeopenres.25127.r93590

**Published:** 2024-09-02

**Authors:** Hiroshi Wada

**Affiliations:** 1 University of Tsukuba, Tsukuba, Japan

This is a valuable report of the genome of *A. irregularis*. Because I am not an expert of the genome analysis, I make comments from the aspect of echinoderm biology. Technical issues should be judged by other experts of genome analysis.

 But actually, although the authors provided some biological information in the Background section, they describe no implication they found from the genome. I like to see some biological implication the authors draw from the genome. Why do they like to sequence the genome of this species?

 Some information should be provided for the individual the DNA was extracted. Is the individual male or female? Which tissue DNA was extracted from?

Are sufficient details of methods and materials provided to allow replication by others?

Yes

Is the rationale for creating the dataset(s) clearly described?

Yes

Are the datasets clearly presented in a useable and accessible format?

Yes

Are the protocols appropriate and is the work technically sound?

Yes

Reviewer Expertise:

Embryology and evolutionary biology

I confirm that I have read this submission and believe that I have an appropriate level of expertise to confirm that it is of an acceptable scientific standard.
